# Flash Smelting Copper Concentrates Spectral Emission Measurements

**DOI:** 10.3390/s18072009

**Published:** 2018-06-22

**Authors:** Luis Arias, Sergio Torres, Carlos Toro, Eduardo Balladares, Roberto Parra, Claudia Loeza, Camilo Villagrán, Pablo Coelho

**Affiliations:** 1 Electrical Engineering Department, Universidad de Concepción, Concepción, CCP 4070386, Chile; sertorre@udec.cl (S.T.); pcoelho@udec.cl (P.C.); 2 Metallurgical Engineering Department, Universidad de Concepción, Concepción, CCP 4070386, Chile; ctoron@udec.cl (C.T.); eballada@udec.cl (E.B.); rparra@udec.cl (R.P.); claudialoeza@udec.cl (C.L.); cvillagranf@udec.cl (C.V.)

**Keywords:** spectral measurements, optical sensors, smelting, copper, sensing, two-wavelength method, optical pyrometry

## Abstract

In this paper, we report on spectral features emitted by a reaction shaft occurring in flash smelting of copper concentrates containing sulfide copper minerals such as chalcopyrite (CuFeS_2_), bornite (Cu_5_FeS_4_) and pyrite (FeS_2_). Different combustion conditions are addressed, such as sulfur-copper ratio and oxygen excess. Temperature and spectral emissivity features are estimated for each case by using the two wavelength method and radiometric models. The most relevant results have shown an increasing intensity behavior for higher sulfur-copper ratios and oxygen contents, where emissivity is almost constant along the visible spectrum range for all cases, which validates the gray body assumption. CuO and FeO emission line features along the visible spectrum appear to be a sensing alternative for describing the combustion reactions.

## 1. Introduction

Nowadays, the application of optical methods to evaluate industrial processes, involving chemical reactions, is a prominent field of research and development. This is because the use of appropriate optical sensors combined with well-defined measurement methods can provide important spectral information, which can be related to several process conditions to be evaluated. In several processes, such as combustion of hydrocarbon fuels, spectroscopy techniques are particularly suitable because of the non-contact nature of the optical sensing technology to capture the flame’s spectral emissions. Further, optical passive or active sensors such as laser-based diodes, CCD cameras, radiometers, photodiodes, photomultipliers and UV cells provide information such as flame instability, O_2_ concentration, CO pollutants emission, flame geometry, energy distribution, identification of molecules, atoms, radicals and ions, and also concentration variation of these elements with temperature and operational conditions [1,2,3,4,5,6].

The copper pyrometallurgy industry faces many challenges. To achieve major energy efficiencies, environmental compliance and desired products composition, a proper feedback from the process becomes an essential and a difficult task to accomplish due to the harsh sensing environment. In this context, optical techniques appear to be a suitable alternative. Optical sensing of metal combustion also provides on-line process monitoring main variables to improve the metal recovery technique.

In particular, one of the most important processes involving chemical reactions in copper mining occurs in the flash smelters, aiming to recover copper from copper concentrates [7,8,9]. In this process, the concentrate is injected through a burner, producing a suspension of solid particles into an oxygen-enriched chamber. Individual particles heat up as they flow down through the combustion chamber and after ignition they start to combust producing light pulses into the oxygen-enriched environment. This process takes place in the so-called reaction shaft [10]. It is in this zone where heterogeneous reactions take place, leading to liquid and gas phase’s generation. These reactions produce very fast light pulses and they release energy radiating heat and light. Until now, these reactions have been well-modeled using physics, chemistry and heat-transfer fundamental laws, introducing models to handle the complications of two-phase flow of particles taking into account the effect on the burner regulation [11]. These mathematical models have created a comprehensive knowledge of the burners and reaction shaft combination, helping to understand dust formation and reaction rate combustion [12,13]. However, due to inaccuracies about concentrate properties and oxygen combinations, the predictions achieved by such proposed models imply errors and uncertainties, requiring sensors to validate such models and to characterize the real operational conditions.

The core of this work is dedicated to identify visible band spectral features, from intensity calibrated spectral measurements, to recognize the proper operation of a flash smelting copper concentrates process. To up to our knowledge, and after an exhaustive literature search, there are no spectral measurement reports of the emitted reactions, in the visible (VIS) band, from the shaft smelting of copper concentrates. To measure such spectral emissions is relevant in order to better understand how operational conditions can be related more accurately with results of the combustion process. Particularly, we outline spectral features emitted by flash smelting of copper concentrates containing sulfide copper minerals such as chalcopyrite (CuFeS_2_), bornite (Cu_5_FeS_4_) and pyrite (FeS_2_). These spectral features are fundamental to develop new optical tools to improve the operational conditions of the flash smelting stage in the Chilean copper industry.

This paper is organized as follows. Section 2 is focused on brief descriptions of main radiometric issues, from the signal point of view, regarding the typical spectral features normally seen in industrial process measured spectrum of radiating shaft. In Section 3, spectral measurements are shown carried out in a laboratory set up drop-tube arrangement, and in a real copper smelting plant, identifying several spectral features present in copper concentrates combustion. In Section 4, temperature and spectral emissivity of copper concentrates, burned at high temperature, are estimated. Finally, concluding remarks are given.

## 2. Spectral Signal Considerations

In general an industrial process that is spectrally studied exhibits typical emitted spectral features, which should be separately analyzed to discover their correlation with the process operational conditions. From the signal analysis point of view, spectral features from a process are typically classified in terms of discontinuous and continuous (or baseline) spectrum, depending on the spectral distributed energy along the studied spectral band. Discontinuous features are typically characterized by a high frequency behavior over narrow spectral bands, with defined center-wavelength (CWL) and full-width at half maximum (FWHM), whereas continuous features commonly exhibit small frequency behaviors on a wide spectral band [14]. Discontinuous features are typically associated with atomic and molecular emissions, which depending on the CWL and FWHM provide information about the fundamental components presented in the reactions. However, continuous emissions are typically associated with incandescent particles generated in the reactions, exhibiting closely related blackbody emission at high temperature. A typical example can be seen in hydrocarbon and biomass-based flame spectra, where the emitted energy in the VIS band exhibits discontinuous radiations like CH* and C_2_* radicals, centered at 432 nm and 516 nm respectively, with a typical FWHM of 10 nm, and also atoms emissions like Na and K, centered at 588 nm and 769 nm respectively, with a FWHM of 3 nm. All these features are added to a continuous spectral background related to incandescent soot particles, emitting at high temperatures [1,14]. Both continuous and discontinuous features each provide key information associated to the process production performance.

Formally, in real spectral measurements, the spectral infinite dimension is reduced to a set of N finite channels. Let us consider that the measured spectral radiance I*_m_*(λ,*T*) is emitted by a shaft in a flash smelting process, where λ ∈ [λ*_min_*,λ*_max_*] is the wavelength and *T* is the shaft temperature. A shaft emitted sampled spectrum can be represented as I*_m_*(λ*_i_*,*T*), where *i* = 1, ..., *N* and λ*_i_* ∈ [λ*_min_*,λ*_max_*] for all *i*. Therefore, a measured shaft spectrum I*_m_*(λ*_i_*,*T*) is composed by the sum of continuous I*_c_*(λ*_i_*,*T*), and discontinuous spectrum I*_d_*(λ*_i_*,*T*), that can be expressed as:

(1) $$I_{m}(\lambda_{i},T)=I_{c}(\lambda_{i},T)+I_{d}(\lambda_{i},T).$$

## 3. Copper Concentrates Spectral Measurements

In this section, the laboratory experimental set up to measure the spectra emitted by copper concentrates is described. Measurements achieved in this set up and in an industrial copper smelting setting are discussed.

### 3.1. Experimental Setup

**Figure 1:**
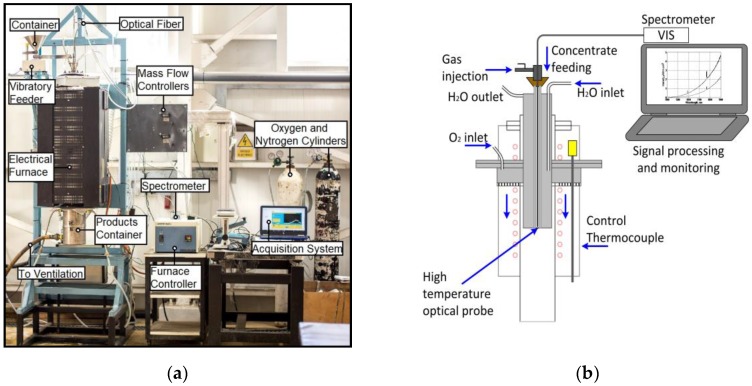
Experimental setup: (**a**) Experimental setup picture depicting main components; (**b**) Optical setup of a constructed pinhole to shaft spectra access.

The experiments were carried out in a flash smelting laboratory prototype formed by a drop-tube arrangement heated with an electric resistance. This arrangement is provided with a controllable vibratory feeder of copper concentrate particles, a controllable oxygen flux, thermocouples and a dust and gas collector. A high-temperature isolated optical fiber (Avantes Ltd., Apeldoorn, The Netherlands) was installed inside of a nitrogen refrigerated pinhole, also aimed to feed the concentrate and the oxygen. With such setup, the reaction zone of the shaft was spectrally measured, covering the whole field of view of the fiber. An USB4000 spectrophotometer, previously calibrated with a HL-2000-CAL lamp for irradiance measurements in units of µW/nm cm^2^ (booth manufactured by OceanOptics Inc., Largo, FL, USA), was used to collect the spectra [15]. With such a device, a 350–1100 nm band was measured with a resolution of ~0.22 nm. Figure 1a shows the laboratory scale setup arrangement, and Figure 1b shows the details of the optical pinhole constructed to access the reaction zone of the shaft.

Emitted spectra from three predominant chalcopyrite CuFeS_2_ concentrates were measured, each of them composed with a different S/Cu ratio. An S/Cu ratio of 1.07 for concentrate A, an S/Cu ratio of 1.27 for concentrate B, and an S/Cu ratio of 1.85 for concentrate C. The copper concentrates where provided by Chagres Mining (Anglo American), located in San Felipe, V Region of Chile. In Table 1, the mineralogical composition of the concentrates is summarized, measured with a QEMSCAN^®^ (Quantitative Evaluation of Minerals by SCANning electron microscopy, Fei Company, Hillsboro, OR, USA), while in Table 2, concentrates’ main proportion of composed elements, such as S, Cu and Fe. Values in Table 1 and Table 2 are indicated in percentage by weight units, wt.%. Additionally, traces of lead and zinc sulfides, but in fewer quantities, were found, besides calcium aluminum oxides.

The concentrates were introduced into the drop-tube and burned at different oxygen conditions from 30%, 45%, and 60% to 80%. The combustion chamber was heated at 773 K to facilitate concentrate ignition. After ignition, a natural combustion is generated by shaft temperature.

During the concentrate flash oxidation with the oxygen, many chemical reactions may occur. Some of these main identified reactions are summarized in Table 3 with the respective calculated standard enthalpy of formation, $\Delta H_{f}^{o}$ values at 298.15 K. The importance of these reactions lies in the possibility to correlate resultant molecules with the spectral features presented in the measured spectrum.

Depending on the experimental conditions, the concentrate compounds may present varying degrees of reactivity. For example, in general higher temperatures and oxidizing conditions are conducive to a higher degree of sulfides reaction, for thermal decomposition and for the subsequent formation of oxides. Now, we proceed to depict our spectral measurements.

### 3.2. Spectral Measurements and Analysis

**Figure 2:**
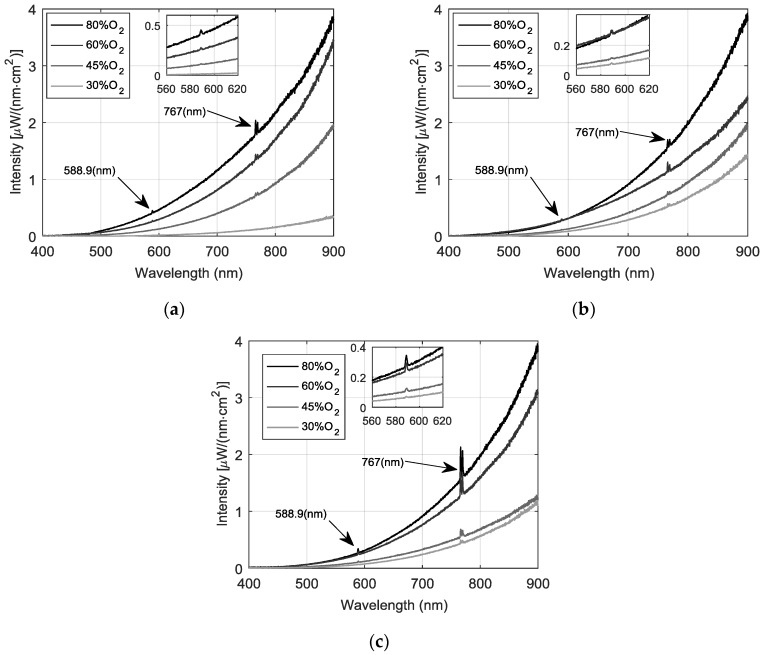
Emitted spectra during the flash smelting process, for three different copper concentrates with a: (**a**) S/Cu ratio of 1.07; (**b**) S/Cu ratio of 1.27 and (**c**) S/Cu of 1.85.

In Figure 2, the measured calibrated spectra are depicted, obtained under the mentioned conditions above. Since measurements were stable during the experiments, only one average spectral signal is reported under each oxygen condition at each specific S/Cu ratio. Both, continuous and discontinuous spectral features, produced by the concentrate smelting, are measured for the shaft radiation. The most likely molecular band emissions are from the excited states of metal oxides or metal hydroxides formed by these metals in the presence of the oxygen flow. First, note that the continuous baseline follows a blackbody emitting pattern, showing an increasing intensity when the oxygen in the drop tube is raised. Indeed, higher signal discrimination is achieved over 650 nm, making the reddish spectral band appropriated for following the oxygen effect over the combustion process. Further, this black body behavior is related to an increment in the particle combustion temperature produced by the increasing gas oxygen content. The higher oxygen content also resulted in a change of mechanism from relatively constant combustion temperatures to more rapid transient combustion pulses in a pure oxygen process. Note also that increasing the S/Cu ratio from concentrate A to C raises the spectrum intensity at lower oxygen concentrations. In other words, the sulfur mass in the solid reaction characterizes the particle reaction rate, and it can be said that when the amount of sulfur oxidation increases, the continuous spectral intensity increases as well, since such reactions are highly exothermic (e.g., reaction 10, Table 3); hence, increasing the release of heat with less oxygen. In the next section, the two-wavelength method is implemented to online temperature estimation in order to corroborate the temperature behavior.

Please note that two predominant discontinuous radiations peaks can be observed, one centered at 588.9 nm and the second, a double-peak around 767 nm. In all cases, as expected, the intensities of the discontinuous radiation increase with the amount of injected oxygen. The first spectral line centered at 588.9 nm could be associated with the presence of Na, which arises even at small quantities, because of its high transition probability. Notwithstanding the foregoing, and due to the high presence of Fe and O reacting into the shaft, this discontinuous radiation is more likely to be associated to a molecule of FeO [16], according to the flash reaction four in Table 3. This dilemma was fully discussed in [16] experimentally showing that both Na and FeO emit a very similar discontinues peak around 588 nm.

Spectral copper emissions were absent entirely. This was quite unexpected for the copper study, as copper is known to exist primarily in the free atomic form during combustion. It was not so unexpected for the magnesium and aluminum studies. Magnesium, an alkaline earth metal, is known to exist primarily in a molecular form in combustion.

The second double peak centered at 767 nm follows a typical feature, which is produced by K atoms. Similar to the Na atoms, this element is presented in small quantities; however, it possesses a high transition probability. Other authors have reported these line emissions as common molecular interferences during combustion [17], and so in this work they are ignored in subsequent analysis.

### 3.3. Spectral Measurements in An Industrial Copper Smelting Plant

**Figure 3:**
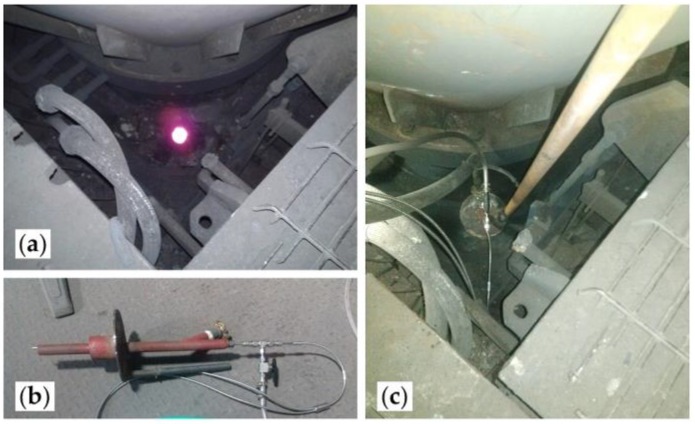
Industrial measurement setup: (**a**) smelter top peephole; (**b**) steel cooled protecting probe and cooled optical fiber; (**c**) mounted probe.

The technique was also implemented in a real industrial flash smelting plant (Chagres Flash Smelting Plant, Anglo American, Catemu, Chile). To capture the spectral data, the optical fiber, protected by an air cooled steel probe, was introduced through one of the top peepholes located near the furnace feeder, Figure 3, and this allowed the acceptance cone of the fiber to be directly focused towards the flame. Due to difficulties related to real operations conditions, only two spectral measurements are shown in this paper. 

**Figure 4:**
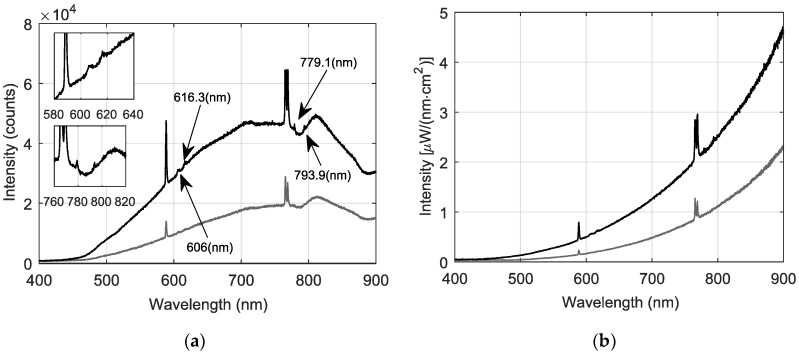
Emitted spectra during the flash smelting process in a flash smelter (real plant): (**a**) Uncalibrated spectra; (**b**) Calibrated spectra.

Figure 4 depicts the measured uncalibrated and calibrated spectra obtained under the conditions mentioned above. Please note that the Cu_x_O band appears at 606 nm and at 616 nm. Our results are similar to the ones reported in [18] for copper alloys used in the space shuttle main engine [19]. This occurs because, under oxidizing conditions, Cu tends to form CuO as well as metal. When this happens, the Cu_x_O dissolves in the slag generated during the making of cooper. Also the peaks at 779.1 nm and 793.9 nm correlate strongly with Fe emissions from the NIST (National Institute of Standards and Technology, U.S. Department of Commerce): Atomic Spectra Database website [20]. Please note that the preceding spectral features did not show up in our laboratory set up measurements. This is explained because at the industrial level, flames emit radiation from a greater population of excited and stable species (refer to Maxwell-Boltzmann distribution [21]) than flames at a laboratory scale. Also, different flame sensed regions may have been assessed, which may change the sensed spectral profiles, as depicted in [14].

## 4. Temperature and Emissivity Estimation

First, the estimation of the combustion temperature using a spectral technique is implemented, and then, emissivity is calculated. As the combustion process radiates mainly continuous spectra, the two-wavelength method is better suited for such a task, due to similarities with the spectral behavior of blackbody radiation described by Planck’s law [22,23,24]. To employ this method, the spectral radiance I in radiometric units, µW/(nm∙cm^2^), at two different wavelengths I_1_ = I(λ_1_) and I_2_ = I(λ_2_), should be selected. Then, the temperature of the process can be estimated as follows:

(2) $$\hat{T}=\frac{c_{2}\left(\frac{1}{\lambda_{1}}-\frac{1}{\lambda_{2}}\right)}{\ln(R)+5\cdot \ln\left(\frac{\lambda_{1}}{\lambda_{2}}\right)-\ln\left(\frac{\varepsilon (\lambda_{1},T)}{\varepsilon (\lambda_{2},T)}\right)}$$

where *c*_2_ = 1.438 × 10^−2^ (m∙K) is the second Planck’s constant, *R* = I_1_/I_2_, and *ε* is the emissivity at each wavelength at certain temperature. Please note that (2) is derived from Wien radiation law, hence it is valid only for λ_1_, λ_2_ ≤ 1000 (nm) and processes with temperatures *T* ≤ 3000 K. This approximation is also used for the later emissivity estimation. Additionally, since emissivity may vary with wavelength and temperature, it is always desirable to find the pair λ_1_, λ_2_ where $\varepsilon (\lambda_{1},\hat{T})/\varepsilon (\lambda_{2},\hat{T})\approx 1$, i.e., emissivity at both wavelengths varies in the same proportion at any temperature and exhibits a gray body emission behavior. To choose the wavelength pair that fulfills this assumption, in this paper we evaluate first the wavelength interval Δλ that minimizes the dispersion $\sigma_{\hat{T}(\Delta \lambda )}$ of estimated temperature values, as described in [24,25]. The next procedure describes the proposed solution to this problem, which is-based in an exhaustive search.

**Figure 5:**
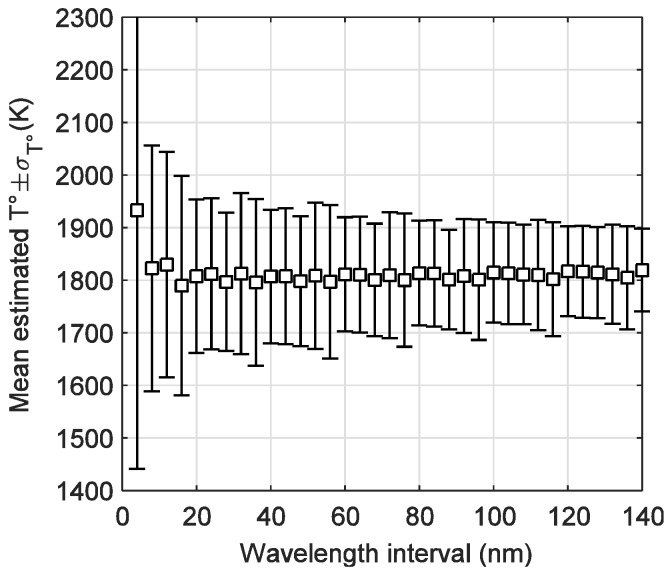
Mean estimated temperature by using two wavelength method and standard deviation as a function of wavelength interval Δλ.

By starting from a value λ_1_, a Δλ = λ_2_ − λ_1_ was defined for each 4 nm; i.e., Δλ = [4, 8, …, 150] nm, avoiding line or broad band emissions. Thus, the shaft temperature was calculated using (2), with different wavelength pairs combinations at each Δλ, starting from λ_1_ = 500 nm, and for each experiment the starting wavelength is increased in 20 nm. In this work, the intensity profile for the concentrate B with a 60% O_2_ and a constant emissivity is taken as a sample profile to find out the proper wavelength pair and wavelength interval. In Figure 5 the calculated combustion temperature is depicted. Please note that as Δλ increases, less dispersion is achieved, meaning that for the estimated shaft temperatures, emissivity at both wavelengths tends to be constant, reaching almost the same temperature average (2024 K) and dispersion (*σ*_T_ = 114 K) from about Δλ = 100 nm. 

To determine proper starting wavelength λ_1_, for each Δλ, the wavelength that achieves the closest estimated temperature to the average $\mu_{\hat{T}(\Delta \lambda )}$ is selected as the most appropriate, i.e., the goal is to find λ_1_ such as the difference $\left|\hat{T}(\lambda_{1},\Delta \lambda )-\mu_{\hat{T}(\Delta \lambda )}\right|$ is minimized. By performing this analysis, it was found that for all Δλ this value ranged from λ_1_ ≈ 640 nm to 660 nm for most of the cases. Then, a wavelength interval of Δλ = 100 nm and starting wavelength of λ_1_ = 650 nm are used (λ_2_ = 750.1 nm) for final temperature estimation. Although these values are proposed as best for calculations, in this work, results are reported with percentual estimation errors between estimated temperatures and mean temperature values (assumed as reference and calculated as the preceding analysis at selected Δλ) for each case. The results are summarized in Table 4. It can also be seen that percentual errors range between 0.8% and 7.9%, which agree with results reported in literature where the two-wavelength method is used. Please note that temperature values are also in agreement with the theory, laboratory experiments and operation of flash smelting furnaces [26,27,28,29]. Additionally, by applying the preceding procedure, the estimated combustion temperatures of industrial signals depicted in Figure 4 are 1803.0 K (▬ signal) and 1995.0 K (▬ signal) respectively.

Once temperature has been estimated, the spectral emissivity can be retrieved by comparing measured spectral radiation I*_m_*(λ,*T*) with blackbody emission B(λ,*T*) described by Wien’s law:

(3) $$B(\lambda ,T)=\frac{c_{1}\cdot \exp\left(-c_{2}/\lambda T\right)}{\lambda^{5}}\left(10^{-7}\cdot \mu W/\left[\mathrm{nm}\cdot {\mathrm{cm}}^{2}\cdot \mathrm{sr}\right]\right)$$

where a factor 10^−7^ is used to ensure consistency of units with the measured spectra, and *c*_1_ = 1.176 × 10^−16^ (Wm^2^/sr) is the first Planck’s constant. Then, by using same measuring solid angle Ω*_O.F_*_._ = π∙(*N.A.*)^2^ (sr), with *N.A.* as the optical fiber numerical aperture (= 0.22 in our system), emissivity is calculated as ε(λ,*T*) = I*_m_*(λ,*T*)/(B(λ,*T*)∙Ω*_O.F_*_._) [25,30]. Figure 5 shows the estimated emissivity for each combustion condition.

**Figure 6:**
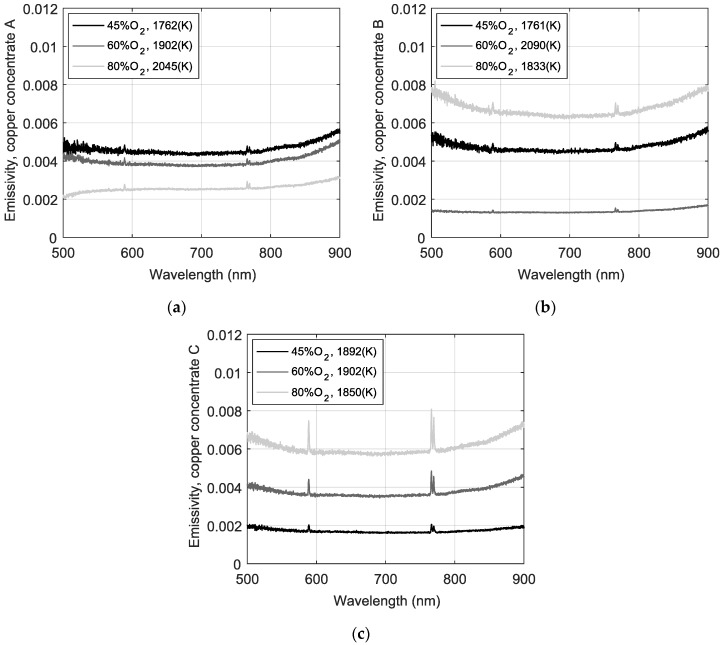
Spectral emissivity during the flash smelting process, for three different copper concentrates of a: (**a**) S/Cu ratio of 1.07; (**b**) S/Cu ratio of 1.27 and (**c**) S/Cu of 1.85.

**Figure 7:**
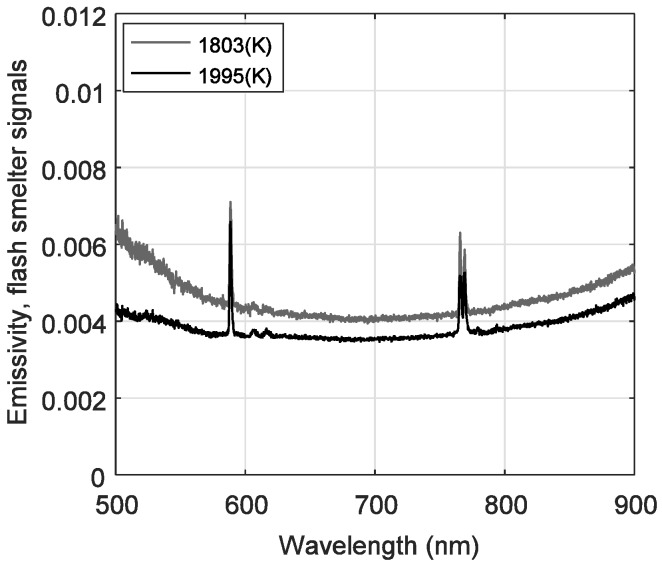
Spectral emissivity during the industrial flash smelting process, for two measurements.

Please note that emissivity curves in Figure 6 are displayed in the wavelength range from 500 nm to 900 nm, since for shorter wavelengths the limited performance of the optical system introduces numerical errors when emissivity is estimated. Moreover, the lower spectra intensities detected at 30% O_2_ condition caused numerical errors during estimation, thus such results are not displayed. It can also be seen from Figure 6 that from 600 nm to ~760 nm the spectral emissivity is approximately constant for all cases and that ε(650 nm,*T*)/ε(750.1 nm,*T*) ≈ 1, justifying the wavelength selection for temperature estimation in the preceding analysis. As a validation test, combustion temperature values were calculated by considering the emissivity profiles showed in Figure 6, and these values were the same depicted in Table 4. These results validate the assumption of constant emissivity during the first estimation. Also, from Kirchhoff’s radiation law, it can be said that since the ignited particles portray a low spectral emissivity along the visible spectral region, which equals the absorptivity of the particles, in thermal equilibrium most of the radiation is scattered out from the cloud. On the other hand, for increasing wavelengths towards the infrared region, the emissivity seems to monotonically increase for all cases, which accounts for a strong thermal radiation absorption behavior of copper concentrate particles in that spectral region. The same behavior is observed in Figure 7 for emissivity curves estimated from the spectra measured in the industrial flash smelter.

Finally, it can be seen that the spectral emissivity profiles, derived from the proposed methodology, present a non-linear behavior for S/Cu ratios approaching the unity, and a remarkable linear behavior for a copper concentrate with S/Cu = 1.85 regarding the %O_2_.

It is important to highlight that the variability on the mineralogical characteristics of copper concentrate is a natural perturbation to the system. Thus, the implications of this findings and proposed procedures will allow the development of different models to predict these perturbations and to support the operation of Cu smelters. In future work we plan to develop an instrument and an experimental procedure for conducting proper industrial scale measurements. Also, it would be of major interest to model an emissivity function that properly characterizes the radiation emitted by the cloud of copper concentrate particles in terms of physical properties such as particle size distribution.

## 5. Conclusions

In this work, calibrated spectral measurements from a laboratory scale setup as well as from a real copper flash smelting process are reported. The results show that the measured and retrieved information presents spectral features that could be useful for improving copper concentrates combustion performance. Spectral features for CuO, and possibly for FeO sensing, were outlined, as well as others such as spectral intensity variation with copper-sulfur ratio and oxygen excess. From a conceptual perspective, the validation of the gray body emission behavior of copper concentrate in the visible spectral range from 600 nm to ~760 nm at high temperatures, becomes essential for proper temperature estimation with the two-wavelength method. Also, a methodology for wavelengths selection is presented. On the other hand, although some spectral emission lines appears as useful features for describing the copper flash smelting process, in most of the cases they are masked out by the continuous radiation background, limiting its usefulness for real industrial applications.

## Figures and Tables

**Table 1:** Concentrates mineralogical composition.

Concentrate	A (wt.%)	B (wt.%)	C (wt.%)
CuFeS_2_	89.17	67.44	61.23
Cu_5_FeS_4_	0.89	2.13	1.24
FeS_2_	5.08	17.71	31.23
CuS	2.00	7.02	3.26
FeO	0.46	0.44	0.61
Cu_2_O	0.02	0.06	0.05
MoS_2_	0.28	0.41	0.05
SiO_2_	1.90	2.05	2.03

**Table 2:** Concentrates main proportional composed elements.

Concentrate	S (wt.%)	Cu (wt.%)	Fe (wt.%)	S/Cu Ratio
A	32.25	30.27	27.55	1.07
B	34.86	27.47	26.51	1.27
C	38.74	20.89	32.28	1.85

**Table 3:** Typical reactions during concentrate flash oxidation ^1^.

Reaction		$\Delta H_{f}^{o}$ (kJ/mol)
1	CuFeS_2_ = 0.5Cu_2_S + FeS + 0.25S_2(g)_	81.10
2	Cu_5_FeS_4_ = 2.5Cu_2_S + FeS + 0.25S_2(g)_	112.06
3	FeS_2_ = FeS + 0.5S_2(g)_	134.13
4	FeS + 0.5O_2(g)_ = FeO + 0.5S_2(g)_	−101.30
5	CuS + 0.5O_2(g)_ = CuO + 0.5S_2(g)_	−35.50
6	Cu_2_S + O_2(g)_ = 2CuO + 0.5S_2(g)_	−167.80
7	Cu_2_S + 0.4FeS + 0.1S_2(g)_ = 0.4Cu_5_FeS_4_	−44.83
8	CuO + FeO = CuFeO_2_	−89.89
9	2FeO + SiO_2_ = 2FeO·SiO_2_	−33.72
10	S_2(g)_ + 2O_2(g)_ = 2SO_2(g)_	−722.23

^1^ A $\Delta H_{f}^{o}<0$ value indicates the reaction is exothermic (releases thermal energy), whereas a $\Delta H_{f}^{o}>0$ value indicates an endothermic reaction (absorbs thermal energy). Values are calculated with HSC Chemistry^®^ (Outokumpu Research Oy, Pori, Finland).

**Table 4:** Copper concentrates estimated temperature ^1^.

Concentrate	Temperature (K) and (Estimation Error in %)			
	O_2_ = 30%	O_2_ = 45%	O_2_ = 60%	O_2_ = 80%
A	1655 (7.9)	1762 (2.6)	1902 (3.0)	2045 (3.8)
B	1732 (4.3)	1761 (2.2)	2090 (3.8)	1833 (1.9)
C	1754 (1.9)	1892 (0.8)	1902 (2.4)	1850 (1.9)

^1^ Temperature estimation considering constant emissivity.
